# A Comparative Study on the Outcomes of Hypospadias Surgery Following Early Versus Late Bladder Catheter Removal

**DOI:** 10.7759/cureus.26104

**Published:** 2022-06-20

**Authors:** Anurodh Kumar, Ishwar Ram Dhayal

**Affiliations:** 1 Urology and Renal Transplant, Dr. Ram Manohar Lohia Institute of Medical Sciences, Lucknow, IND

**Keywords:** tip repair, tubularized incised plate urethroplasty, snodgrass repair, meatal stenosis, urethrocutaneous fistula, early catheter removal, hypospadias

## Abstract

Background

Hypospadias is the most common penile malformation affecting up to one in 300 live male births. In general, a urinary diversion (urethral stent or bladder catheter) is maintained in situ for two to seven days or more after hypospadias repair. Because of the low level of evidence, the latest guidelines of the European Association of Urology and the European Society of Paediatric Urology provide no recommendations concerning the timing of catheter removal after hypospadias surgery. In this study, we aimed to compare the outcomes of hypospadias surgery (tubularized incised plate urethroplasty, TIPU) following early versus late bladder catheter removal.

Methodology

In total, 62 patients were included in this study. All patients underwent TIPU by the same team of surgeons. All patients were divided into the following two groups: group A (32 patients) had their catheter removed on or before the fifth postoperative day, and group B (30 patients) had their catheter removed after the fifth postoperative day. All patients were scheduled for an outpatient assessment after two weeks, at one month, after three months, and at six months if necessary.

Results

The mean age of patients in group A was five years (three to seven years) and in group B was five years (four to 7.25 years) with a p-value of 0.378. Among the early complications of the surgery, the occurrence of wound infections, urinary tract infections, and urinary retention was comparable among the two groups. The rate of bladder spasms (0% versus 13.3%, p = 0.033) was significantly higher in group B than in group A. The rate of urinary retention (12.5% versus 0%, p = 0.045) was significantly higher in group A than in group B. Superficial wound infection occurred in two out of 32 patients in group A (6.3%) and two out of 30 patients in group B (6.7%) (p = 0.94). Both groups had similar incidences of wound complications. Urinary tract infections also had a similar incidence in both early and late catheter removal groups, i.e., one out of 32 patients in group A (3.1%) and three out of 30 patients (10%) in group B (p = 0.271). Urinary extravasation following hypospadias repair occurred in two out of 32 patients (6.3%). No extravasation was noted in the late catheter removal group. However, the difference was not clinically significant (p = 0.164). Two patients in both groups developed urethrocutaneous fistula (6.3% in group A versus 6.7% in group B). However, the difference was not clinically significant. Meatal stenosis developed in three out of 32 patients in group A and two out of 30 patients in group B (9.4% versus 6.7%; p = 0.696). One patient in the early catheter removal group developed urethral stricture as a late complication. None of the patients in the late catheter removal group developed this complication.

Conclusions

The occurrence of long-term complications of TIP hypospadias repair was not affected by the early removal of the bladder catheter. The shortcomings of our study were its descriptive nature and the small sample size. Further prospective randomized controlled trials are needed to ascertain the safety of early catheter removal and improvement in quality of life in the immediate postoperative period.

## Introduction

The incidence of hypospadias is one in 300 live newborn male children making it the most common malformation affecting the penis. Its etiology is mostly unknown and multifactorial [1]. Hypospadias has the following three sets of anomalies of the phallus: urethral meatus may be situated on the undersurface of the penis from the glans to the perineum; the penis has a ventral bending termed chordee; and the foreskin is mostly deficient ventrally coupled with the anomalous distribution of the foreskin dorsally known as the “hood” [2]. Treatment goals in hypospadias are categorized into two parts, namely, curvature correction and good caliber neourethral creation, referring to a distally located slit-like neo-meatus on glans apex resulting in even coherent unobstructed urinary stream producing good cosmesis and improving the self-confidence of the child. In this process, the anatomy and physiology of the penis become normal, and the procedure has minimal complications. Hypospadias is a unique congenital pathology with hundreds of described surgical procedures, and history is replete with these surgical aspects and technical reviews [3-7].

Most surgeons divert the urine post-procedure either using a urethral stent or bladder catheter for 2-14 days depending on the individual severity of the case. There are very few studies on catheter-less urethroplasty and evaluation of its safety and efficacy in the literature. The European Association of Urology (EAU) and the European Society of Paediatric Urology (ESPU) do not provide any specific guidelines regarding catheter removal post-procedure due to low level of evidence [8]. Removal of the catheter immediately post-procedure can decrease the discomfort of both the patient and relatives leading to early discharge. However, removing the catheter early may raise concerns about the occurrence of urethrocutaneous fistula and wound dehiscence due to the flow of urine across the still healing suture line. Conversely, prolonged catheter drainage may increase the risk of wound infection, meatal stenosis, and urethral stricture.

In this study, we aimed to evaluate the effects of the timing of catheter removal after hypospadias surgery on the occurrence of the aforementioned complications and the overall success of the surgery. This knowledge would help us in defining the appropriate timing for catheter removal after surgery to minimize unnecessary delay without increasing the risk of complications.

## Materials and methods

This prospective, observational, descriptive study was conducted in the Department of Urology and Renal Transplant at a tertiary-level referral hospital in northern India. The study duration was 18 months from December 2018 to June 2020. All cases of penile hypospadias with ages >six months operated within the study duration were included in the study. Patients with syndromes, major congenital malformations, psychiatric illness, neurological/non-neurological bladder dysfunction, associated upper tract changes, altered renal function, and redo cases were excluded. The study was approved by the Institutional Ethics Committee of Dr. Ram Manohar Lohia Institute of Medical Sciences (IEC no. 77/18).

Procedure

Detailed and relevant medical history was taken, a thorough examination was done, and all patients were investigated according to the study protocol.

Tubularized Incised Plate Urethroplasty (TIPU)

All patients underwent TIPU, the technique described previously by Snodgrass et al. [9]. The procedure was done by the same team of surgeons. Standard degloving of the penis was performed, and to ascertain curvature an artificial erection test was done in all cases. For significant curvature, dorsal plication of the tunica albuginea was performed. The urethral plate was incised up to the depth of buck’s fascia in the midline, and an appropriately sized urethral catheter (6-12 Fr) was used. The neourethra was tubularized over it in standard Snodgrass fashion. A 6-0 monofilament polydioxanone suture material was used in all reconstructions, and neourethral coverage with standard dartos flap was universally used to strengthen the repair. A non-compressive dressing was applied for 96 hours.

All patients were divided into two groups: group A (32 patients) had their catheter removed on or before the fifth postoperative day. Group B (30 patients) had their catheter removed after the fifth postoperative day.

All patients were scheduled for an outpatient assessment after two weeks, at one month, after three months, and at six months if required. Various parameters such as age at surgical repair, hypospadias type, chordee association, and the timing of catheter removal were recorded. Early complications such as bleeding, wound infection, bladder spasm, painful micturition, urinary retention, and urinary extravasation were noted. The definition of urine retention was considered to be a patient with palpable bladder with pain. Positive urine culture with associated symptoms was indicative of urinary tract infection. Late complications included the occurrence of meatal stenosis, urethral stricture, and urethrocutaneous fistula (UCF). UCF and meatal stenosis were diagnosed by the surgeon during the follow-up. The median follow-up time for both groups was six months. All parameters between the two study groups were compared and recorded.

SPSS version 21.0 (IBM Corp., Armonk, NY, USA) was used to evaluate the various parameters. Assessment of numerical data from non-normal scattering was demonstrated as the median (Q1-Q3), and the Mann-Whitney test was used for analysis. Other statistical tests such as Pearson’s chi-square test, Fisher’s exact test, and two-sample proportion tests were employed for comparison of categorical data. P-values of <0.05 were considered significant, and all p-values were two-tailed.

## Results

This study included a total of 62 patients categorized into two groups: group A included 32 patients and group B included 30 patients.

Baseline parameters of the two groups

In this study, the mean age of patients in group A was five years (three to seven years) and in group B was also five years (four to 7.25 years). Significant chordee was found in 25% of patients in group A and in 16.7% of patients in group B. Glanular, coronal, and distal penile hypospadias were present in 68.8% of patients in group A versus in 63.3% of patients in group B. Mid and proximal penile hypospadias was present in 31.3% of patients in group A versus in 36.7% of patients in group B. All baseline parameters were comparable between these two groups (Table 1).

**Table 1:** The baseline parameters of the two groups. Age is represented as median (Q1–Q3).

	Group A, number (%)	Group B, number (%)	P-value
Number of patients	32 (51.6%)	30 (48.4%)	
Age (years)	5 (3–7)	5 (4–7.25)	0.378
Chordee	8 (25.0%)	5 (16.7%)	0.417
Hypospadias site			
Distal	22 (68.8%)	19 63.3%	0.652
Mid/Proximal penile	10 (31.3%)	11 (36.7%)	

Early Complications after the procedure in the two groups

Among the early complications of the surgery, the occurrence of wound infections, urinary tract infections (UTIs), and urinary retention was comparable among the two groups. The rate of bladder spasms (0% versus 13.3%, p = 0.033) was higher in group B than in group A. Urinary retention rate (12.5% versus 0%, p = 0.045) was quite high in group A than in group B (Table 2).

**Table 2:** Early complications after the procedure in the two groups.

Complications	Group A, number (%)	Group B, number (%)	P-value
Wound infection	2 (6.3%)	2 (6.7%)	0.94
Urinary tract infection	1 (3.1%)	3 (10.0%)	0.271
Bladder spasm	0 (0.0%)	4 (13.3%)	0.033
Urinary retention	4 (12.5%)	0 (0.0%)	0.045
Urinary extravasation	2 (6.3%)	0 (0.0%)	0.164

Late complications after the surgery in the two groups

Two patients in both groups developed urethrocutaneous fistula (6.3% in group A versus 6.7% in group B); however, the comparison was not clinically significant. Meatal stenosis developed in 9.4% of the patients in group A versus 6.7% of patients in group B; however, the comparison was statistically insignificant. One patient in group A developed urethral stricture as a late complication. Among the patients in group B, none developed this complication (Table 3).

**Table 3:** Late complications after the surgery in the two groups.

Complication	Group A, number (%)	Group B, number (%)	P-value
Urethrocutaneous fistula	2 (6.3%)	2 (6.7%)	0.94
Meatal stenosis	3 (9.4%)	2 (6.7%)	0.696
Urethral stricture	1 (3.1%)	0 (0.0%)	0.32

## Discussion

Creating a functional neourethra and aesthetically acceptable normal phallus is the main goal of hypospadias repairs. Snodgrass repair is increasingly being favored by a large number of surgeons, especially in the last two decades [10-13]. There is a continuous debate regarding catheter or urethral stent use in hypospadias surgeries. Maintaining dry anastomosis is the main logic for bladder drainage. Bladder spasms and detrusor muscle contractions produced by foreign bodies due to bladder wall irritation are the factors against the use of urethral catheters or stents.

Buson et al. [14] reported complication rates of 18.9% and 4.6% in non-stented and stented groups, respectively, and recommended a catheter or stent in these repairs. Although UCF incidence is minimized by using a catheter, there is a slight increase in patient discomfort [15]. Another study by El-Sherbiny [16] concluded that patients with distal hypospadias could be operated on with no stent in toilet-trained children; however, postoperative complications were lower when a stent was used for one week. The study indicated that a catheter is a good idea, especially in toilet-trained children.

On the contrary, there are reports showing that unstented repair results in better healing. For normal epithelialization after neourethral construction, the use of a stent is not required and urine flow may help in keeping the healing edges separate allowing the regeneration of the urothelium [17]. In an interesting study by Almodhen et al. [18], it was reported that postoperative care is better in patients with no urethral stents, and simultaneously the need for medications such as antibiotics and anticholinergics may be omitted. In a retrospective setting, Leclair et al. [19] compared patients with stents with unstented Snodgrass repair and found no difference in outcomes. In a case series of 41 patients, Turial et al. [20] concluded that patients with distal and mid-penile hypospadias can be managed without the use of a urethral stent in Snodgrass repair without any complications and improved postoperative comfort.

Snodgrass [9] recommended urethral stents for five to seven days. He rationalized that this duration helps in improved healing and prevents complications. On the contrary, many studies have concluded that seven-day stenting results can be obtained in one to three days or in an unstented setting as well. Rich et al. demonstrated that long-term results with overnight stenting for patients with distal and mid-penile hypospadias were similar to reported data [19]. In animal studies [16-21], it has been shown that in Snodgrass repair tissue healing starts by re-epithelialization of the incised plate. For partial re-epithelialization five days and for complete epithelialization and normal urothelium two weeks are required [16]. Thus, removal of the catheter on day one would not lead to complications such as stenosis or strictures. Strictures occur due to concentric healing of the incised edges as opposed to re-epithelialization. These findings indicate that resumption of early urinary flow in the neourethra may help in keeping the healing edges separate supporting early catheter removal and minimizing stricture formation.

Radwan [22] demonstrated that late catheter removal had a higher rate of meatal stenosis (12.7%) than early catheter removal during the first three days (0%). In our study, early removal of the catheter was not related to increased immediate morbidity. The rates of wound infection and UTIs were comparable in both early and late catheter removal groups. Thus, prolonged catheterization (>five days) did not contribute to an increased rate of wound infections or UTIs.

Urinary retention, despite occurring in the early catheter removal group, was uncommon and further management was easy. Overall, 12.5% of patients developed urinary retention in our study; however, compared to the study by El-Sherbiny (24%) [16], it was much lower and comparable to boys after circumcision surgery in a routine setting. Four patients had postoperative urinary retention with a painful palpable bladder in the early catheter removal group. Recatheterization was done, and suprapubic cystostomy was not required in any of these patients. The use of 2% lidocaine jelly was associated with lubrication and helped in anesthetizing, and all these patients tolerated the catheter well. Copious lubrication coupled with a graduated centrally deep relaxing incision which was quite shallow proximally helped in preventing the development of false passage while inserting the catheter [16]. There were no long-term complications in these children.

Moreover, the development of bladder spasms was significantly decreased in the early catheter removal group compared to the group that had an indwelling catheter for >five days (0% versus 12.5%, p = 0.045). Similar rates of bladder spasm were reported by Xu et al. in their study of non-stented versus stented TIP repair (0% versus 10.7%). Anticholinergics were not required in the early catheter removal group. There was a subjective improvement in comfort in the early catheter removal group. Most patients who had a prolonged indwelling catheter were quite irritable until the catheter was removed.

Urinary extravasation occurred in two out of 32 patients (6.25%) in the early catheter removal group. Xu et al. reported a rate of 1.3% for urinary extravasation in non-stented TIP repair. Both these patients developed urinary extravasation on postoperative day two after catheter removal. Both patients were managed by recatheterization. The catheters were later removed on postoperative day five. One out of these two patients later developed a urethrocutaneous fistula. The association between urinary extravasation and urethrocutaneous fistula was not significant.

The incidence of urethrocutaneous fistula was similar in both groups in our study (6.25% versus 6.66%). In a study by Hashim et al., early removal of urethral catheter significantly reduced the occurrence of UCF following TIP repair (6.6% for the group with catheter removal on postoperative day one in comparison with the other group in which one-third developed fistulae after stent removal on postoperative day six). Our study also supports the notion that the early removal of catheters has no impact on the occurrence of UCF. Meatal stenosis rates in our study were 9.37% for group A compared to 6.66% for group B. Both were comparable indicating that early removal of catheter did not significantly alter the occurrence of meatal stenosis. Radwan showed a significant difference regarding meatal stenosis between early removal of a urethral catheter within two to three days (0%) and patients who had a long urethral catheterization period (12.7%) [23]. Similar results were also reported by Hashim et al. wherein 13.3% of patients with catheter removal on postoperative day one had meatal stenosis against 38.7% of postoperative day six catheter removal patients. In a retrospective study of 254 patients with distal and mid-penile hypospadias who underwent Snodgrass repair, Xu et al. demonstrated that catheter duration is not associated with stenosis and showed that catheter-less Snodgrass repair is possible and positive outcomes can be obtained without increasing complications and simultaneously taking care of patient comfort [24].

In our study, one out of 32 patients in the early catheter removal group developed stricture (3.1%); however, no patients developed stricture in the late catheter removal group. Moreover, the occurrence of urethral stricture was found to be independent of the duration of indwelling catheter.

Limitations of the study

The limitations of our study included its descriptive nature and the small sample size.

## Conclusions

There is no significant difference in the incidence of early and postoperative complications between the removal of the stent before or after the fifth postoperative day. However, early catheter removal patients experienced a subjective improvement in comfort, and prolonged indwelling catheter patients were more irritable until the catheter was removed. Further prospective randomized controlled trials are needed to ascertain the safety of early catheter removal and improvement in quality of life in the immediate postoperative period.
